# A Multi-Sensing IoT System for MiC Module Monitoring during Logistics and Operation Phases

**DOI:** 10.3390/s24154900

**Published:** 2024-07-28

**Authors:** Husnain Arshad, Tarek Zayed

**Affiliations:** Department of Building and Real Estate, The Hong Kong Polytechnic University, Hong Kong; husnain.arshad@connect.polyu.hk

**Keywords:** modular integrated construction, IoT, sensors, safety, structural health monitoring, logistics

## Abstract

Modular integrated construction (MiC) is now widely adopted by industry and governments. However, its fragile and delicate logistics are still a concern for impeding project performance. MiC logistic operations involve rigorous multimode transportation, loading-unloading, and stacking during storage. Such processes may induce latent and intrinsic damage to the module. This damage causes safety hazards during assembly and deteriorates the module’s structural health during the building use phase. Also, additional inspection and repairs before assembly cause uncertainties and can delay the whole supply chain. Therefore, continuous monitoring of the module’s structural response during MiC logistics and the building use phase is vital. An IoT-based multi-sensing system is developed, integrating an accelerometer, gyroscope, and strain sensors to measure the module’s structural response. The compact, portable, wireless sensing devices are designed to be easily installed on modules during the logistics and building use phases. The system is tested and calibrated to ensure its accuracy and efficiency. Then, a detailed field experiment is demonstrated to assess the damage, safety, and structural health during MiC logistic operations. The demonstrated damage assessment methods highlight the application for decision-makers to identify the module’s structural condition before it arrives on site and proactively avoid any supply chain disruption. The developed sensing system is directly helpful for the industry in monitoring MiC logistics and module structural health during the use phase. The system enables the researchers to investigate and improve logistic strategies and module design by accessing detailed insights into the dynamics of MiC logistic operations.

## 1. Introduction

Hong Kong is adopting the modular integrated construction (MiC) method to address concerns about labor shortages and construction sustainability. The offsite manufacturing of modules offers more control for better management of environmental emissions and has proven to be more cost- and time-effective. Over 75 MiC projects have been initiated in the last few years, some of which have been completed successfully [1]. Despite its largely reported success, some critical challenges have been related to its supply chain and logistic operations [2]. For Hong Kong, the primary motivation for MiC adoption is the just-in-time (JIT) module arrival and immediate assembly to avoid storage of modules and other construction-related congestion. However, several supply chain uncertainties hinder JIT assembly, such as (a) the number of stakeholders having conflicting goals and interests [3], (b) fragmented supply chain segments functioning independently [4], (c) multimode, cross-border transit [5], (d) strict geometrical and dimensional constraints for transportation and assembly, (e) uncertain storage requirements, and (f) uncertain site assembly [6].

Meanwhile, an ambidextrous MiC supply chain (SC) requires a push-flow of modules to ensure seamless assembly, while a strict assembly sequence requires a demand-based pull flow of the modules [7]. In such a scenario, any assembly delay would snowball the whole supply chain, causing unwanted on-site accumulation of modules, poor site space management, extensive accumulated inventories, and excessive storage costs [3]. Such cost damages are also evident in some recent cases in Hong Kong [5,8]. The external factors of assembly delays, like weather conditions or transportation regulations, can be managed with better planning and scientific predictions. However, assembly delays due to module safety and structural health are more uncertain and critical. The MiC assembly process involves several supervision checks to ensure the module’s safety and check for any damage. If damage is found, the module is repaired on-site or sent to the workshop for major repair, causing additional assembly delays [9]. Also, on-site physical inspection can only look into visible damage, and any latent cracks in the module could be overlooked, causing serious safety issues during crane lifting. Moreover, the structural members prone to damage are often hidden behind drywalls or fireproofing. Therefore, their physical inspection is not possible.

In prefabricated modules, damage is initiated mainly during logistics operations, that is, transportation and module handling [10]. Godbole et al. [11] studied the impact of acceleration on a module during transportation and found that vertical acceleration can reach up to 32 m/s^2^ (3.3 g). However, damage does not only occur due to vertical acceleration; horizontal shocks due to instant braking and road roughness may also severely impact the module. Similarly, the impact in the form of strain due to stacking of the module during storage and loading-unloading operations can also cause damage to the module. Such damage may also cause misalignment of modules for assembly, causing further delays while repairing such issues. Thus, it is vital to continuously monitor the module structure during the assembly process to ensure safety and avoid any hazards. Also, the continuous cyclic impact of logistic operations may induce latent damage to the module, appearing later during the building use phase in the form of cracks, leaks, etc. That may reduce the overall service life of the module. Therefore, early damage detection is critical for the safety and long-term performance of the structure.

To monitor the MiC module’s damage, safety, and structural health, it is essential to measure its structural response continuously. However, the MiC module generates a non-stationary structural response during highly dynamic logistic operations. Monitoring such non-stationary structures is highly challenging, where both the structure and the impacting loads are moving [12]. Most existing technologies and methods for damage and structural health monitoring (SHM) are designed for traditional stationary structures [13]. The structure of a traditionally constructed building is mostly monolithic, where the structural response at any location on the building can be sensed or estimated from any other location. However, in the case of MiC, the building comprises separate building blocks (modules), where damage in one module cannot be detected from any other module, as they are not joined monolithically. Therefore, several sensors must be installed on each module individually to monitor each module’s structural response and performance.

The most commonly used sensing technologies for SHM are (a) vibration-based, (b) strain-based, (c) guided waves, and (d) acoustic emissions [14]. Table 1 summarizes the sensors and features of each sensing technology. Acoustic emission and guided wave technologies follow an active signal response estimation principle [15]. A short pulse/signal is induced in the structure, and the sensors installed at different locations sense the response. The variation in the sensor’s measured response leads to an estimate of the variation in the structural condition. These techniques are considered suitable for mid-range assessment and require sophisticated equipment and a static environment for signal induction. Thus, using these technologies for MiC module structure monitoring during highly dynamic and non-stationary logistic operations is unsuitable.

On the other hand, strain gauge and vibration sensors do not require any standard signal induction, and they measure variation in the structural response under different environmental and loading conditions. Strain gauge sensors can directly estimate the structural deformation or displacement locally. Meanwhile, variations in the vibration response can help assess global structural changes. Also, linear vibrations and rotational speed variations effectively capture structural movement, which can help estimate the impact of loadings induced by the motion. A multi-metric sensor containing an accelerometer and gyroscope would be beneficial for monitoring such motion. Since module lifting, loading-unloading, and assembly operations involve the tilt and rotation movement of the module, its impact on the structure and corresponding response must be monitored [16].

Considering the sensors’ sensitivity, range, and portability, the accelerometer for vibration, gyroscope for rotational speed measurement, and strain gauges are most suitable for monitoring MiC modules during logistic operations. However, commercially available accelerometer and strain sensors are not integrated and have separate control, support, and communication systems, such as computers, wireless gateways, and battery or power supply. Each MiC module requires a dense array of sensors to monitor logistic operations effectively. Installing several large commercially available sensing systems on a single MiC module is impractical.

Recent advancements in the Internet of Things (IoT), sensing technologies, and microcontrollers have enabled the development of integrated sensing systems. Following this, Spencer et al. [17] developed modular-type sensor boards (nodes) for acceleration and strain measurement. Each node has a different sensor or module connected to each other to make a fully functional sensing system. Fu et al. [18], Won et al. [19], and Sarwar et al. [20] expanded this system and demonstrated different application scenarios for monitoring bridges and precast structures. These application scenarios highlighted that the system has lesser portability, a larger size, and higher power consumption. It has no sensors to measure rotational speed or tilt, which is essential for monitoring MiC logistic operations [16]. Furthermore, it requires a PC-based base station closer to the monitoring site for real-time data acquisition. More recently, Khayam et al. [21] developed a similar sensing system for monitoring the lifting of prefabricated girders. This system adopts more advanced MCUs and analog-to-digital converters (ADCs) to enhance strain measurement efficiency. However, the installed accelerometer range (±2 g) is not enough to measure the impact of transportation scenarios. Also, the system lacks real-time data transmission and relies only on built-in SD card storage.

The MiC module requires the installation of several sensing units on each module to monitor the module structure effectively. Thus, the form factor of the sensing units is critical. Visibly large sensing units installed on the module may attract the attention of building occupants and cause interruption. However, the size of previously developed sensing systems was significantly higher and less desirable for MiC modules. Also, MiC logistics monitoring requires long-range communication to ensure the real-time monitoring of modules during transportation from remote areas. Previously developed systems lack such long-range real-time data transmission capabilities.

Considering the above-discussed criticality of MiC logistics and the limitations of existing sensing systems, there is a dire need to develop an integrated, multi-sensing device to continuously monitor the module’s structural response throughout its logistic operations and the building use phase. Also, the sensing devices should be compact enough to install on a module and have real-time wireless communication capabilities. Monitoring the module’s structural response at each instance during its SC can help predict its structural health and safety before each upcoming operation, thus allowing decision-makers to make timely decisions and avoid assembly disruptions. Also, such comprehensive monitoring will enable structural health to be tracked throughout its lifecycle and proactively plan maintenance of the MiC building.

Therefore, in this study, (1) a smart wireless sensing system is developed that adopts microsensing technologies, integrates them in a compact small device that can be easily installed on a module, and enables IoT-based communication; (2) the developed system is tested and calibrated to ensure high precision and accuracy; and (3) a field experiment demonstrates its detailed application for real-time damage assessment and health monitoring of the MiC module during logistics operations. The demonstrated damage assessment and health monitoring methods help identify possible damage in real-time operations.

The paper is organized into a total of six sections. Following the introduction, Section 2 explains the development process and salient features of the IoT sensing system. Section 3 evaluates accuracy and performance of the IoT sensing system through different tests and comparisons. Section 4 elaborates on the setup for applying the IoT sensing system to monitor MiC logistic operations. The damage assessment strategies are also discussed in this section. Section 5 presents the results of MiC logistic monitoring and discusses several damage assessment analyses. Finally, Section 6 presents the conclusions and future recommendations of the study.

## 2. Developing IoT Sensing System

**Figure 1:**
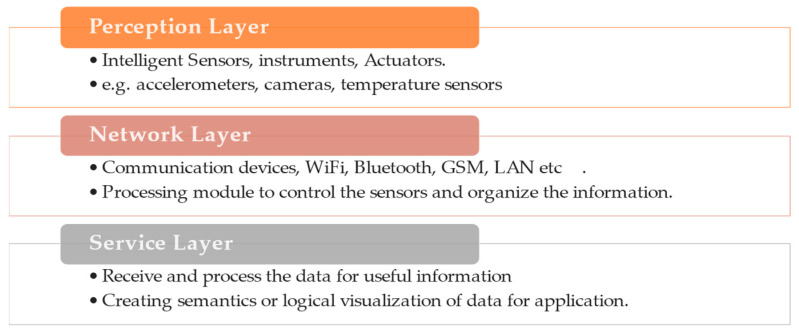
The basic IoT architecture.

The standard IoT system’s architecture consists of three essential layers, as shown in Figure 1 [22]. The first perception layer is the IoT physical node, which consists of intelligent sensors that gather the required information. The second network layer is the active communication layer, which transforms the physically sensed information into organized and logical information and transmits it. This layer stores and processes the received data, presenting it in more a logical form for the application.

**Figure 2:**
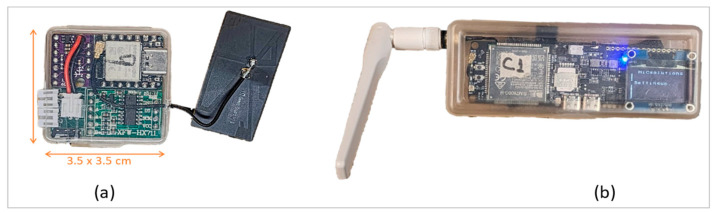
(**a**) Peripheral Sensing Unit (SU), (**b**) Central Communication Unit (CU).

Following this IoT architecture, the developed system comprises peripheral sensing units (SUs) and a central communication unit (CU) representing the perception and network layers of IoT, respectively. The peripheral sensing units are small integrated sensing devices installed over the MiC module structure, as shown in Figure 2. These units monitor the structural strains, acceleration, and tilt angle at several points on the structure. Each peripheral unit is wirelessly synced with the CU and sends real-time data. The central communication unit (CU) then processes all the received data from all the installed SUs, stores data backup, and transmits it to a web server. Further particulars of the developed system are detailed in the following sections.

### 2.1. Peripheral Sensing Unit

The design rationale for the peripheral sensing unit (SU) is based on practical constraints during MiC logistics and building use phases. Each MiC module needs several SUs for effective monitoring of structural performance; hence, a large number of SUs are required for the whole building. Thus, the development cost for SUs is primarily focused, and cheaper available components are utilized. Further, the form factor of SU is kept minimal, making it practically invisible when installed in a module, thus avoiding any interference to or from the building occupants. First, a double-sided printed circuit board (PCB) was designed to ensure a minimum form factor for SU development. The components—microcontroller (MCU), accelerometer, gyroscope, strain gauge analog-to-digital converter (ADC), Wheatstone bridge, a battery, and some connectors—are mounted on the designed PCB for manufacturing the SU.

The Xiao ESP32S3 (Seeed Studio, Shenzhen, China) is used as an MCU to control IMU and ADC functions and further process the data. The Xiao ESP32S3 is a tiny but robust MCU offering a 240 MHz 32-bit LX7 dual-core processor, enabling enough computational power to handle complex machine learning models as well. It supports integrated 8 MB PSRAM and 8 MB Flash, WiFi 2.4, and Bluetooth 5.0 while consuming 108 mA power at peak performance and 14 μA in sleep mode. The LSM6DS3 inertial measuring unit (IMU), containing integrated 3-axis accelerometer and gyroscope sensors, is used. It’s a high-performance, low-noise IMU that consumes 0.42–1.25 mA power while measuring up to ±16 g acceleration and ±2000 dps angular/rotational speed [23].

An HX711 ADC (Avia Semicon, Xiamen, China) is utilized to read signals from two strain gauges. HX711 is a two-channel ADC widely used as a load cell and is a cheaper alternative. This 24-bit signal amplifier converts the strain signal from strain gauges to digital values (0–1023) [24]. A four-wire Wheatstone bridge configuration is required to connect a strain gauge to the ADC. Quarter Wheatstone bridges are configured for each strain gauge, connecting a strain gauge and three 120 ohm resistors in series.

Moreover, to reduce the current noise and improve the sensor readings, three 100 nf capacitors are connected to each component. JST Ph2.0 connectors are used to connect the detachable strain gauges and battery. A 1200 mAh LiPo battery is attached to the circuit and placed in a compact case. The battery capacity can be enhanced depending on the requirements. The overall size of the sensing unit is around 35 × 35 × 15 mm, and it weighs about 160 g.

The SU firmware is programmed using the Arduino IDE. The ESP-NOW wireless communication protocol is employed in firmware for data transmission between SUs and CU, enabling peer-to-peer communication. ESP-NOW is highly suitable for continuous data transmission scenarios as it offers low latency and consumes significantly low power for peer-to-peer communication [25].

### 2.2. Central Communication Unit

**Figure 3:**
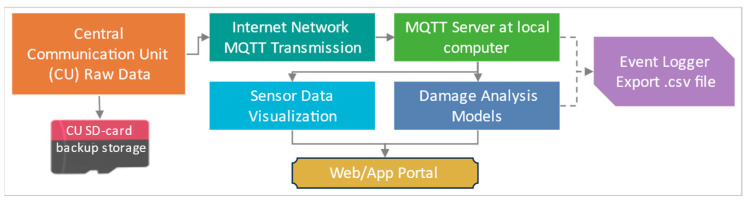
Sensor data communication and storage framework.

The CU is the central unit that connects to all the peripheral units. It can monitor and control the SU’s functioning, such as battery status, switching to low power mode, activating/deactivating any sensor, and requesting data transmission. The primary function of CU is to collect sensor data from all the SUs and transmit that to the server. A built-in module was used to develop CU, which integrates ESP32 MCU (Espressif, Shanghai, China), SIM7600 (SIMCom, Shanghai, China) cellular module, GPS, SD, and WiFi. The CU is also equipped to support large-capacity LiPo batteries and solar charging to enhance its portability. A mini OLED display is attached to the CU to monitor the status of connected SUs and control other functions. ESP32 MCU processes the received data from all the SUs, indexes the data streams, and stores it in the built-in SD card module as a backup. Meanwhile, the SIM7600 module enables real-time data transmission using a 4G internet network, ensuring seamless transmission from remote areas and sites where the availability of WiFi could be an issue. The sensor data communication and storage framework is elaborated in Figure 3.

The CU firmware programming employs the MQTT (Message Queuing Telemetry Transport) protocol for cellular network transmission. MQTT is highly suitable for IoT-based and high-latency networks as it offers lightweight, asynchronous data transmission and can retain the messages in the queue [26]. An MQTT-based server is established on a local computer to receive and log the data. The logged data is conveniently accessible through .txt, .csv, or Excel file formats for further processing and analysis. Additionally, the web server publishes real-time plots of the incoming data to monitor the data visually. Meanwhile, the damage analysis algorithms are programmed in Python, which accesses the sensor data from the server and publishes the results on the web portal.

## 3. IoT Sensing System Performance Testing and Calibration

Different tests and calibrations are conducted to ensure the accuracy of the developed sensing system. The following section explains the evaluation process and results.

### 3.1. Performance Testing

**Figure 4:**
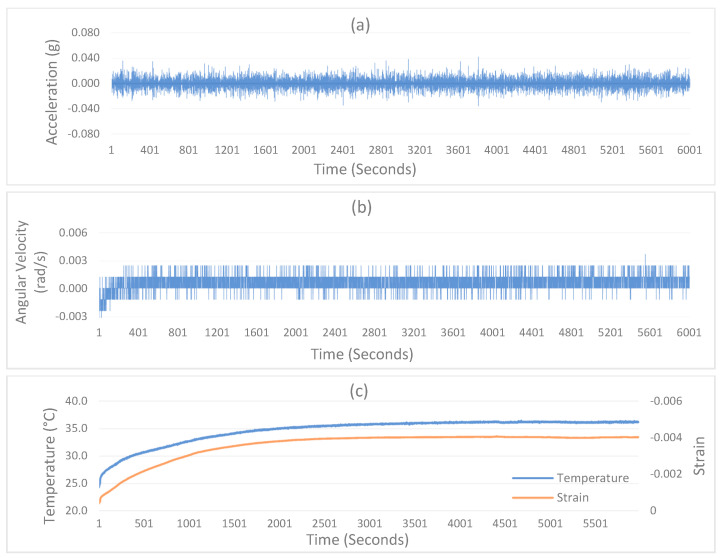
IoT sensing system performance tests under static conditions. (**a**) Acceleration noise, (**b**) Angular Velocity/Gyroscope noise, (**c**) Strain drift under varying temperature.

For performance testing, the SU was placed in a relatively static environment where the 5 m surroundings were restricted to avoid external interference. The readings measured in a static environment represent the noise in the accelerometer and gyroscope, as shown in Figure 4a,b. The 100 min measurements show that acceleration noise in the ±2 g sensing range has a root mean square error (RMSE) of 0.01, mostly between 0.02 to −0.02 g. Similarly, the gyroscope has an RMSE of 0.0023, ranging between 0.003 to −0.003 rad/s. Considering the non-ideal static environment conditions, these results are reasonably comparable with the standard specifications of the LSM6DS3 IMU [23]. In addition to the noise, there is another inherent limitation of any gyroscope, called Turn-On Bias [27]. When a gyroscope is switched on, there will be unstable measurements initially, causing drift and offset [28]. It can be seen in Figure 4b that the gyroscope measurements show some drift in the beginning. To deal with this bias, the SU is programmed to record the unstable measurements at startup and then reduce offset based on initial unstable readings. Thus, the remaining measurements become stable, and the offset is reduced to 0.001 rad/s. Such a minor offset in angular velocity measurements does not affect the relatively calculated rotations and angles.

Further, the strain gauge measurements were tested against temperature variation. For this purpose, the SU was placed in a room where an ambient temperature of 24 °C was maintained. When the SU starts, its components (mainly the MCU) generate heat due to continuous operations. This heat causes the overall device temperature to rise above the ambient temperature until a balance between the ambient temperature and heat dissipation is reached. The time to achieve such a balance is critical for strain measurements, as strain readings are highly sensitive to temperature variations, as shown in Figure 4c. The SU temperature kept growing for the initial thirty minutes and caused the strain values to drift despite no external load being applied. The drift in strain measurement stopped after the balance between the ambient temperature and device heat dissipation was reached, and the temperature was sustained at 36 °C. Similar to the SU’s internal heat dissipation, in real-world scenarios, variations in the surrounding temperature can also cause disruptions to the strain measurements.

### 3.2. Temperature Compensation

**Figure 5:**
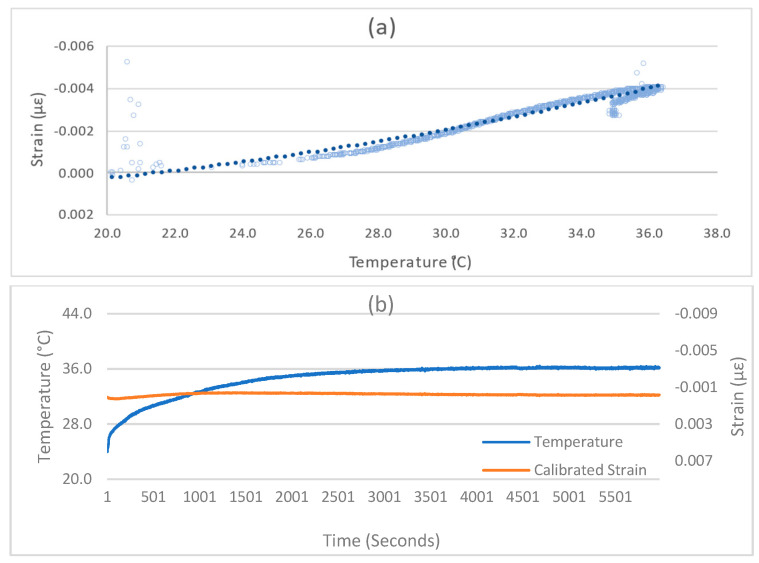
Strain drift and temperature effect compensation. (**a**) Strain Temperature Calibration Model, (**b**) Calibrated Strain.

A model to compensate for the effect of temperature variation is developed to deal with the strain drift issue. The drifted strain values were measured against the varying temperature (24–36 °C) for 100 min. The setup was ensured to be static and vibration-free so that the actual strain remained zero. Then, a second-degree polynomial regression model of drifted strain against varying temperatures was developed, as shown in Figure 5a. The coefficient of determination ($R^{2}$) for the regression model is 0.9397. This regression model gives the calibration factor to further calculate the actual strain values ($S_{a}$), as given in Equation (1), where $S_{d}$ is the drifted strain, and x is the temperature. The actual strain values for this test were calculated using this calibration model and are shown in Figure 5b. It can be seen that the resultant actual strain values have no drift and are now closer to zero, with an RMSE of 0.000254 µε.

(1) $$S_{a}=S_{d}+0.000006x^{2}-0.00008x-0.0011$$

### 3.3. Performance Comparison with UTM

**Figure 6:**
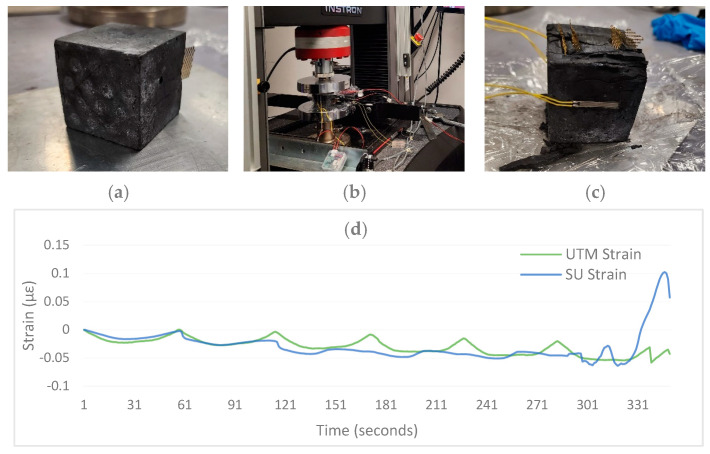
Strain test of the concrete block under cyclic load. (**a**) Concrete block 50 × 50 mm, (**b**) Testing with UTM and SU, (**c**) Cracked block after test, (**d**) Comparision of Strain in Concrete block under cyclic loading.

Finally, the accuracy of the SU is tested by comparing its results with those of a standard universal testing machine (UTM). For this purpose, a compression test under cyclic loading on a concrete block is conducted (Figure 6b). The strain gauges connected to the UTM and SU were installed on opposite sides of the concrete block (Figure 6c). The test results in Figure 6d show that the SU and UTM strain gauges show minor differences in strain values, with just a 0.005 µε RMSE. Then, the concrete block started developing cracks on the SU strain gauge side after the 2nd cycle of loading (115 s). After five loading cycles (300 s), major crack failure occurred, visible in both the SU and UTM strain values. Overall, the test results showed promising performance of SU strain measuring, with a 0.011 µε RMSE, despite early cracks on the SU strain side.

## 4. Application of IoT Sensing System for MiC Logistics Operations

A field experiment was conducted to demonstrate and validate the effectiveness of the developed IoT sensing system. During the field experiment, structure safety was monitored in real time for any potential damage during MiC logistic operations. Besides any critical damage, the overall impact of logistics operations on the module’s structure is also determined, which is helpful for proactive maintenance during the building use phase.

### 4.1. Experimental Setup

The experimental setup was designed to emulate real-world MiC logistic operations. The following sections explain the particulars of the subject module, the sensor installation process, and the observed logistic scenarios.

#### 4.1.1. MiC Module

**Figure 7:**
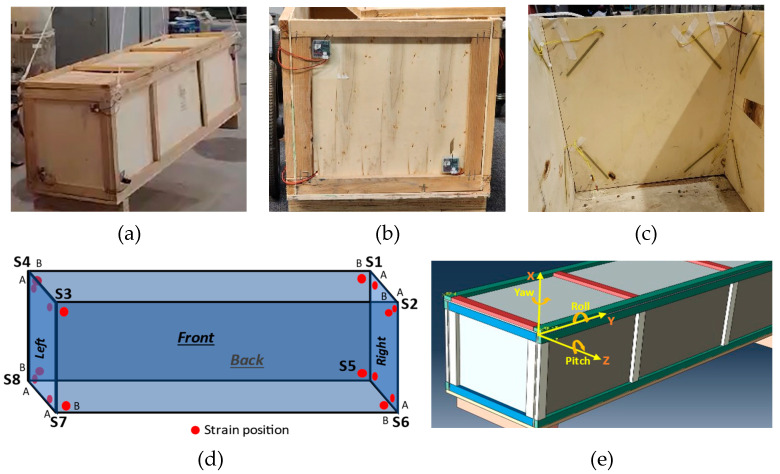
The SU and strain gauge installation on the built wooden module. (**a**) Crane liftin operation, (**b**) SU installation, (**c**) Strain gauge installation, (**d**) Strain gauge locations, (**e**) Yaw, Roll & Pitch rotation axis.

Considering time and cost effectiveness, a small wooden frame-based structure was built to be used as a module. The design of the wooden module was ensured to resemble the actual MiC module structure. The structural frame of this module was built using timber bars having a cross-section of 16 × 36 mm, ensuring reasonable structural strength for the module. The overall dimensions of this module were around 1600 × 500 × 500 mm, having a total weight of around 80 lbs., as shown in Figure 7a,b. The module walls were built using thin balsa plywood with a thickness of 4 mm, whereas the bottom base floor was 16 mm thick. Two timber base supports with a cross-section of 50 × 90 mm were also affixed at the bottom. The properties of the materials used are given in Table 2.

#### 4.1.2. SU Installation

The eight SUs were installed on all corners of the module so that the SUs could sense the whole structure. This arrangement is considered for demonstration and experimental purposes in this study. In other cases, fewer SUs may be installed in selected critical and vulnerable positions on the module. Figure 7c,d highlights the installed SUs’ positions as S1, S2, …, and S8. The SU’s accelerometer will measure the vibrations in three directions: X, Y, and Z. Additionally, the SU’s gyroscope will measure the angular movements in three directions: roll, pitch, and yaw, as shown in Figure 7e. For this study, the accelerometer and gyroscope were set to record measurements at 100 Hz. However, the SU is programmed to transform the data streams into 1 Hz by taking the mean of 100 Hz data. This approach facilitates data syncing, real-time transmission, and managing the quantum of data while ensuring measurement accuracy [29].

Further, each SU can handle two strain gauge sensors installed on adjacent walls at each corner, as highlighted in Figure 7c,d. The 15 cm long foil strain gauge sensors, having a gauge factor of 2, are installed at each wall corner. Such a long strain gauge sensor shall cover a larger corner wall area and sense the maximum strains in the walls. Furthermore, the strain gauge sensors are positioned at 45 degrees at each wall corner, as shown in Figure 7c. The transportation and lifting operations of the module induce critical shear forces in the corners, causing cracks in the walls [11]. Thus, installing strain sensors at 45 degrees will be capable of sensing the maximum possible strain. Hence, the installed strain gauges can sense any deformation anywhere in the structural element. The value of the measured strain will indicate the relative impact at the installed position of the strain and may not directly indicate the damage, but rather the deformation. However, the relative strain impact of all the installed strains can be used to measure and locate possible damage in the structural element.

#### 4.1.3. Logistic Operations

The transportation and crane lifting processes were carried out to demonstrate the MiC logistic operations. For the first 600 s, a crane lifting operation was conducted. Hooks were installed on the four top corners of the module to tie the crane ropes. The module was lifted from the resting platform and hoisted around for a few minutes. During the hoisting process, the module was moved rigorously in all directions to simulate the MiC assembly process. Then, the crane placed the module on a 4-wheel transportation trolley to simulate truck hauling. The module was transported around 200 m away to the final destination. The transportation track involved a rough tile-based track and a relatively smooth asphalt track. Also, it included several turns and inclined surfaces. The transportation speed varied at different points corresponding to the conditions, taking a total transportation time of around 800 s.

### 4.2. Damage Assessment Methodology

**Figure 8:**
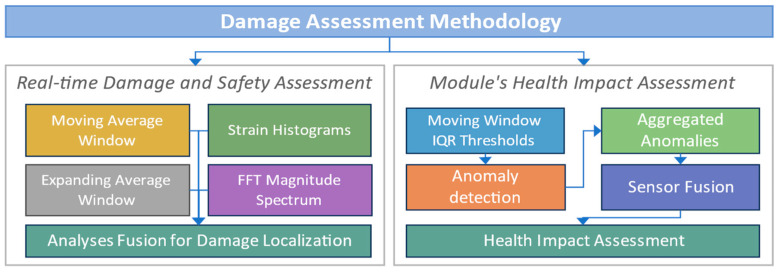
Damage Assessment Methodology.

This study presents various analyses to demonstrate how the sensor-measured response can be used to identify and estimate the damage in the module. These analyses analyze the real-time response of different sensors and evaluate the variations to detect any structural variation, deformation, or damage. The damage assessment strategy has two phases, as shown in Figure 8. In Phase 1, the damage and safety assessment analyses utilizing the real-time sensors’ responses are presented. Different individual analyses are performed for each sensor type to identify the structural variations sensed by it. First, moving average and expanding average windows analyses are performed for strain sensors to determine the damage from the real-time sensors’ response. Then, the strain field histograms and the Fast Fourier Transform (FFT) spectrum magnitudes for the accelerometer and gyro are calculated. Finally, the results of all individual analyses will be compared and fused to confirm and validate the identified damage and assess its location.

Other than critical damage or cracks in the module, there could be undetectable deterioration in the overall module structure caused by the impacts of logistic operations. Such deterioration may reduce a module’s useful life and require early unanticipated maintenance. In the second phase, the sensor fusion approach is adopted to estimate the overall impact on the module’s health. Sensor fusion involves aggregating the relative impact sensed by each installed sensor. The impact is calculated based on the anomalies in the sensor’s response. First, the anomaly detection approach identifies all the significant anomalies in the sensors’ measured response. Then, these anomalies are systematically aggregated for different sensors to calculate the overall impact on module walls. Further details of the damage assessment processes are discussed along with the results in the following section.

## 5. Damage Assessment Results and Discussion

**Figure 9:**
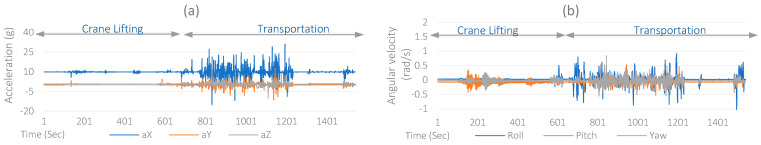
Real-time data trend of (**a**) Acceleration, and (**b**) Gyro/Angular Velocity.

During the field experiment, the IoT system provided a real-time response from all the sensors installed on the module. The real-time sensor response is plotted to analyze the events of logistic operations. The variations in the sensors’ response help estimate the nature of the operation and any significant anomaly in that operation. The acceleration and gyro time series plots, shown in Figure 9, indicate various module movements during crane lifting and transportation operations. The crane lifting operation (between 0–600 s) was slow and smooth; thus, low acceleration variations were observed compared to transportation.

Similarly, the gyro response indicates restricted roll rotation as the module was tied at four corners during the lifting operation. On the other hand, the slight variations in yaw and pitch values during 150 to 300 s indicate the free movements of the hanging module. During transportation, the rough road section is highlighted by the high acceleration and gyro response in all directions from 770 to 1220 s.

**Figure 10:**
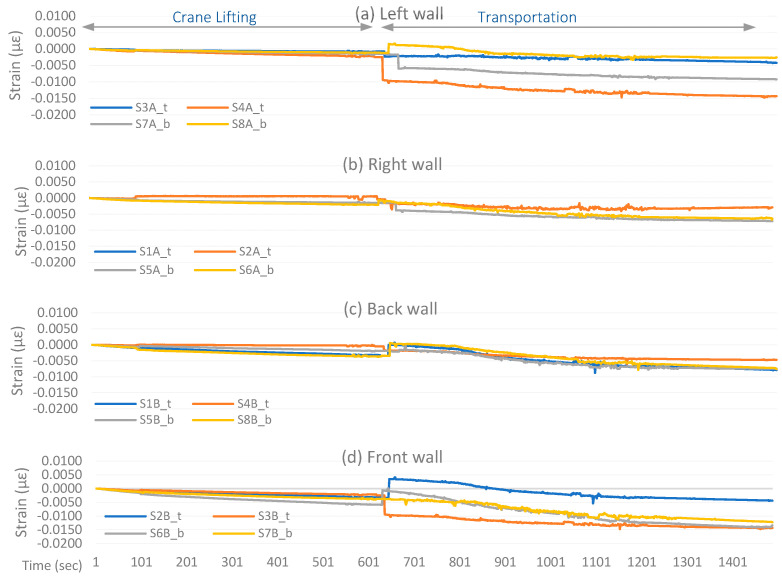
Real-time data trend of strain measurements.

The strain sensors’ real-time response is shown in Figure 10. Despite the rigorous module movements in multiple directions, low strain variation is observed during crane lifting. This was due to the low hanging weight of the wooden module and the relatively smooth lifting operation. The sensors installed at the back and front module walls observed comparatively slight variation. However, these variations reach a maximum of 0.0045 µε, which is insignificant for a wooden module considering its material flexibility and cannot be confirmed as damage without detailed investigation.

Similarly, the strain values observed significant variation at the end of the crane operation as the module was placed on the transportation trolley. Such variation could be due to re-adjusting wooden parts according to the new support conditions, or it may indicate some damage. However, distinguishing such variations as damage requires additional analysis and investigation. Following that, during transportation, some of the sensors observed a slight drift that could indicate damage propagation under the vibrations induced by the rapid movement on the road.

### 5.1. Real-Time Damage and Safety Assessment

Several real-time exploratory analyses are performed to identify potential damage and its location. These analyses can help decision-makers investigate the sensors’ response in detail and assess possible damages while evaluating the relative response of sensors installed at various locations on the module. Further evaluation and comparison of all these analyses confirm the damage and its locations on the module.

#### 5.1.1. Moving Average Window

The general sensors’ response trends visualizations may not be helpful enough to predict damage in the module. Thus, the moving average window (MAW) is further analyzed to investigate the real-time sensor’s response. This analysis represents the mean sensor response of a short period, called a window. This approach reduces the noise in the sensor response and represents actual changes that occurred in the structure [30,31]. A 30-s window is selected so that any point in the plot represents a structural change during that period. Such an approach is highly useful for real-time safety monitoring [32].

**Figure 11:**
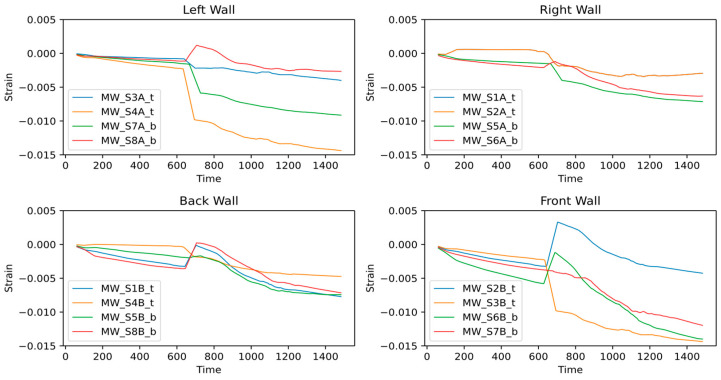
Moving Average Window Analysis.

It can be seen in the moving average window plot, shown in Figure 11, that a significant strain change starts during transportation operations. For the right and back walls, the strain change remains less than −0.008 µε for all the sensors. On the other hand, the left and front walls experienced significant strain changes for most of the sensors attached. The sensors S2B_t show high strain displacement at the beginning of the transportation operation but later return to the average strain trend. The S7A_b sensor shows moderate strain displacement reaching −0.01 µε. The sensors S7B_b, S4A_t, S3B_t, and S6B_b show the most critical response, where strain displacement keeps propagating and reaches up to 0.014 µε.

#### 5.1.2. Expanding Average Window

Like the moving average window, the expanding average window (EAW) calculates the mean strain values. However, instead of using a moving window, all the previous data are considered to calculate the mean strain value for every new point. Such an increasing window size optimally smoothens the window and helps estimate the accumulated variation in the sensor response [31]. Thus, the expanding average window analysis shows a net structural deformation occurring at any plot point.

**Figure 12:**
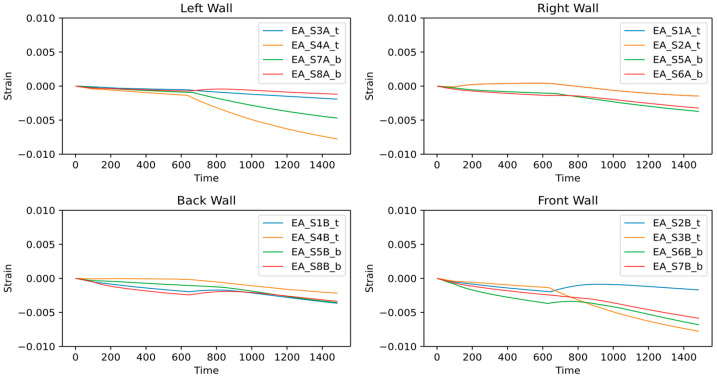
Expanding Window Analysis.

The expanding window plot in Figure 12 highlights critical sensors similar to the moving average window. However, it shows more evident variations in the sensor response and indicates mean net structural deformations. The lines remain horizontal and closer to zero strain, indicating a net-zero deformation in the structure, and lines moving away from zero signify structural deformations. The sensors installed on the right and back walls mostly show nearly horizontal lines closer to zero, thus revealing insignificant deformation in the adjacent walls. On the front wall, three sensors, S3B_t, S6B_b, and S7B_b, show a sharply deviating structural response, indicating evident deformation. Similarly, sensors S4A_t and S7A_b on the left wall show significant deformation.

#### 5.1.3. Strain Field Histograms

**Figure 13:**
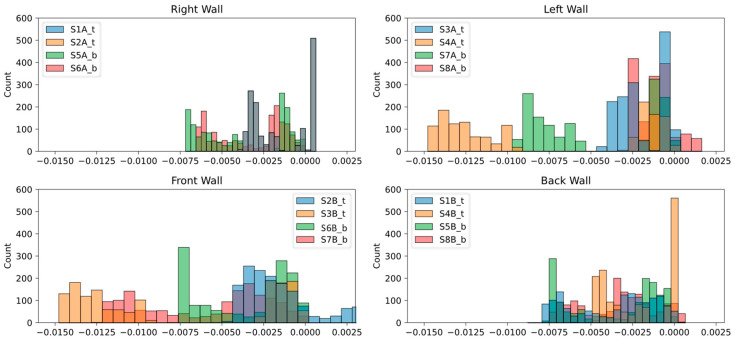
Histograms of Strain Measurements.

The strain field histogram (SFH) helps to compare the frequencies of the discrete strain response values measured over time. Peak strain frequency indicates the amplitudes of various strain measurements, highlighting the variation in the measured response of several installed sensors [33]. Such variations may indicate changes in structural conditions near those sensors [30,34,35]. The SFH plots shown in Figure 13 suggest that the strains’ range or spread is higher in the sensors installed on the left and front walls, reaching up to −0.0150 µε. The sensors installed on the right and back walls measured the maximum strain displacement of around −0.0075 µε. The sensors S4A_t and S7A_b on the left wall and S3B_t and S7b_t on the front wall notably measured an abnormal response compared to other sensors. Also, unlike other sensors, the sensor S2B_t measured abnormal strain values up to 0.0025 µε. The identified discrepancies in the sensor response led to estimating and locating the damage on the module walls.

#### 5.1.4. Fast Fourier Transformation

A Fast Fourier Transformation (FFT) analysis is conducted to evaluate the acceleration and gyro sensors’ response. The FFT magnitude provides the relative strength of various frequency components measured by each sensor. In the FFT spectrum, a distinguished higher magnitude frequency component called the dominant frequency represents the essential characteristics of the logistic operations [36]. In other words, the dominant frequencies indicate the primary structural response under the operations. Thus, if the dominant frequencies of sensors installed at various locations show any variation, it would suggest a change in the structural conditions at that point, i.e., structural damage [32,37].

**Figure 14:**
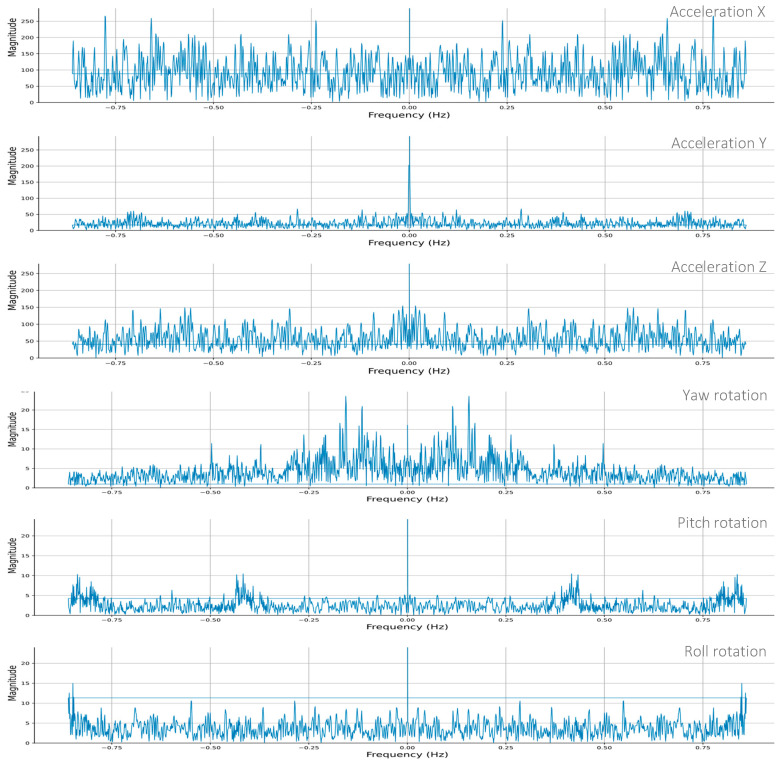
FFT of Acceleration and Gyroscope Measurements—S8.

Figure 14 shows the FFT spectra of acceleration and gyro responses observed for SU-S8. The FFT of each sensor shows multiple dominant frequencies (peaks) representing the non-stationary dynamic characteristics of MiC logistic operations. During all logistic operations, most module movements were along the vertical direction and the shorter side of the module, i.e., the x and *z*-axes, respectively. Thus, acceleration in the x and z-directions has more dominating frequencies. Meanwhile, the y-acceleration has only one distinguished high-magnitude frequency (nearly 0 Hz, called the DC component), representing a dominant average structural response. The modules mostly remained tied along the *y*-axis and did not experience any significant movements along this axis. For the same reasons, the roll rotation has fewer distinguished frequency components for the gyro than the pitch and yaw rotations.

The FFT plots provide complex information, so comparing visualizations may not easily highlight or distinguish any variation. Therefore, all the installed sensors’ interquartile ranges (IQR) are calculated to compare and evaluate the dominant frequencies. The values higher than the 3rd quartile can easily accommodate a signal’s significant dominant frequencies. Similarly, the standard deviation (SD) of an FFT magnitude highlights the spread of the FFT magnitudes across the signal; any considerable variation in SD would indicate a change in structural response near that sensor. Thus, the SD and 3rd quartile can be critical indicators of variation in the structural response [37,38]. Comparing these indicators of sensors installed at different locations can highlight structural change. Table 3 presents the 3rd quartiles and standard deviations (interquartile range) of all the acceleration and gyro sensors.

Along the *x*-axis, the acceleration and yaw rotations do not differ much across different sensors. Due to a complicated dynamic structural response in this direction, it has high noise and several distinguishing FFT magnitude peaks, leading to high SD and 3rd quartile values. Therefore, these sensors may not be suitable for detecting abnormalities. In contrast, along the y and *z*-axes, the acceleration and rotations have comparatively less noise and clear FFT magnitude peaks, thus revealing apparent differences across the sensors. The y-acceleration 3rd quartiles (61.47 a. units) and SD (27.80 a. units) of S1 are significantly higher than those of the other sensors. Similarly, the roll and pitch rotations of S7 and S8 showed minor differences.

To compare the discrepancies systematically and statistically at the locations of different sensors, the normalized impacts of all the 3rd quartiles and SDs are combined by calculating net mean z-scores, as given in Table 3. The highest z-score of S6 (1.03) indicates that the most variation has been sensed near this location, followed by S5 (0.97), S7 (0.90), and S8 (0.87). The 3rd quartile and SD values of acceleration and rotations for these sensors seem significantly abnormal compared to other sensors. Thus, damage is suspected to be closer to the locations of these sensors.

#### 5.1.5. Damage Localization—Analyses Fusion

The analyses above highlight the potential damage in the structure while highlighting the critical sensors that sensed the most abnormal variations in the structural response. As a result, each identified critical sensor could have sensed the same damage from a distant location, or each analysis could have indicated different damage. Therefore, analyses fusion was performed to combine and compare all analysis results to confirm the damage and their respective locations. First, the critical sensors identified by each analysis are categorized into high, moderate, and low categories based on their variation criticality. Sensors in each category indicate that, for instance, the damage is either minor or away from the sensor location. Then, the categorized sensors in each analysis are compared and combined as analyses fusion in Table 4.

The results highlight that the front and back walls experienced high and moderate levels of damage, respectively. On the front wall, the sensors S6, S6B_b, and S3B_t indicate high response variations, confirming critical damage on this wall closer to these sensors. The sensors S7 and S7B_b sensed moderate variations on the front wall, thus indicating the damage location is away from them. Similarly, the S2B_t sensor also sensed a low variation response on this wall, thus indicating the location of critical damage away from it. Considering the locations and relative impact sensed by these sensors, the approximate location of the damage can be estimated using a triangulation approach [39]. The high variations sensed by S6B_b and S3B_t imply the damage location is in the middle of the diagonal between these two sensors. The moderate and low variations sensed by S7B_b and S2B_t suggest that the damage should be a little left and lower than the middle diagonal of S6B_b and S3B_t, as highlighted in the illustration in Table 4.

**Figure 15:**
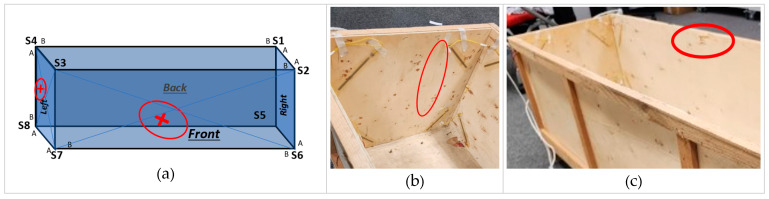
Predicted and actual damage locations. (**a**) predicted damage, (**b**) damage in left wall, (**c**) damage in front wall.

On the left wall, the S4A_t sensor sensed a high response variation, indicating significant damage on this wall. Similarly, the sensors S7 and S7A_b, installed on the bottom right corner of this wall, also sensed moderate response variations. S8 sensed low variations at the bottom left corner of this wall. Now, triangulating the relative impact sensed by each of these sensors, the approximate location of the damage is predicted, as illustrated in Table 4. The back and right walls did not experience any significant damage. Only FFT analysis highlighted sensors S5 and S8 attached on the corners of the back and right walls. However, variations in these sensors are confirmed to be related to the front and left walls. The above-identified damage on the module walls can also be realized in the actual module, as shown in Figure 15. Due to the wooden material of the module, they are hard to see visually. The damage and location predicted on the left wall are similar to the actual damage in the module. However, the predicted location of damage to the front wall is lower than the actual location. This assessment variation is possibly due to the module’s loosely fixed top roof plane during the experiment, which interrupted the response of the sensors installed at the top corners.

### 5.2. Module’s Health Impact Assessment

**Figure 16:**
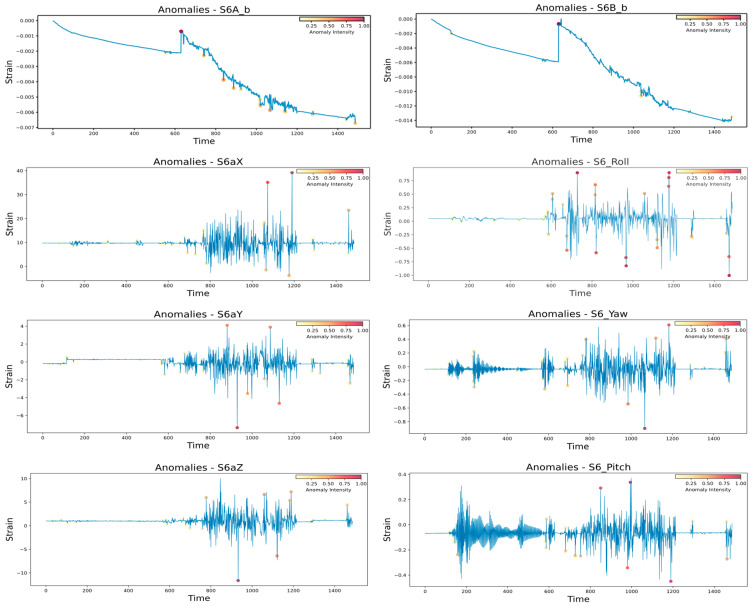
Detected anomalies (as red dots) by sensor S6.

Besides any critical cracks or damage in the module structure, some hidden, intrinsic underlying minor latent damage could remain undetectable. Such minor damage is induced in the structure due to rigorous MiC logistic operations and can further propagate into critical damage during the building use phase. Therefore, it is essential to assess the overall impact of logistic operations on the health of the module structure. Such an assessment can help devise a proactive maintenance schedule for the module and improve the module’s useful life. The adopted approach exploits the typical anomaly detection approach, as all the abnormal logistic impacts are accumulated to calculate the relative impact over different module parts. For anomaly detection, any sensor response in a moving window exceeding the defined threshold is identified as an anomaly [32,40]. Considering a 30-s moving window and one SD (standard deviation) as a threshold, all the anomalies sensed by each sensor were detected. A programmed model detected all the anomalies in real time during the logistic operations, as shown in Figure 16.

The weight of each anomaly during logistic operation is assessed and categorized as high, moderate, and low according to their relative weights. Anomalies are systematically aggregated to calculate a total weighted sum of anomalies for each category at each module wall. Different types of sensors may have varying accuracies when measuring the structural response. Therefore, the impacts experienced by strain, accelerometer, and gyro are also compared individually by comparing the average impacts for each wall and considering equal weights for all types of sensors. The results assessed by the accelerometer suggest different patterns of impacts on the module walls.

In contrast to the previous real-time safety assessment and strain-assessed impacts, the accelerometer assessed the highest impact on the right wall (17.52%) and no impact on the left wall. The impacts on the front and back walls also vary according to the strain sensor assessment. Such variation could be due to the high precision of the strain sensor for assessing closer impacts in contrast to the accelerometer, which can also assess the response from farther locations. Thus, the strain gauge should be considered more relevant for evaluating significant local damage, such as cracks or deformations. However, the acceleration-based assessment could be more useful when assessing the overall structural changes during the stationary building use phase.

The gyro-based impacts followed the strain-based impacts and real-time safety and damage assessment results. However, the estimated values are much lower than the strain-assessed impacts. This is because gyro assessment is based on rotational movements rather than assessing direct local impacts. However, the pattern similarity of strain and gyro-assessed impacts signifies that the rotational movements affected the module structure more than the vibrational movements. Further, comparing the impacts experienced by different types of sensors can lead decision-makers to combine the impacts of different sensors and evaluate the total average impacts on the module. The equally weighted average of all the sensors’ impacts was aggregated. Overall, the front and right walls experienced the most impact, 8.79% and 8.70%, respectively. Significantly, the right wall did not experience any critical damage, as assessed in the previous section. However, the anomalies assessed on this wall highlighted the possible effect on the overall health of this wall compared to the left wall.

Table 5 compares the total, high, moderate, and low impacts experienced by different module walls. The front and right walls are the most affected by the high-level anomalies, which is also evident from the critical damage detected in the previous real-time assessment. Furthermore, the back wall experienced a moderate impact (10.89%), as assessed by the strain sensors. Notably, the right wall did not reveal any critical damage in the previous assessment, but it experienced significantly high anomalies affecting its overall health (9.35%).

## 6. Conclusions

The study embraces the real-time monitoring of the module’s structure during MiC logistic operations. A smart, integrated, portable, IoT-based sensing system is designed to ensure its practicality for MiC logistics. A smaller form factor of sensing units is achieved to keep it practically invisible while installed on a module. The developed sensing system was calibrated by incorporating temperature compensation factors and turn-on bias elimination. The sensing system’s performance is thoroughly tested under different conditions, and its accuracy is found to be comparable to that of standard commercial equipment like UTM.

The module’s real-time structural condition monitoring enables early damage detection, allowing timely decisions to avoid supply chain disruptions. Also, it can improve the on-site safety inspection process while providing more insights into the module’s structural condition, increasing inspection speed, and highlighting the latent damages. Moreover, the safety of the real-time assembly process can be monitored. The sensing system provides detailed structural response data of logistic operations, which is useful for predicting the module’s structural creep and forecasting maintenance during the building use phase. Thus, the system helps to ensure the JIT supply chain for MiC assembly, enhances assembly process safety, and helps to improve the module’s service life.

The application of the developed sensing system is demonstrated with a field experiment, and various analyses are presented to detect critical damage and assess the overall impact on the module’s health. The demonstrated field experiment not only evaluated the system’s effectiveness but also highlighted the effectiveness of different sensors in assessing the structural condition. The strain sensors are found to be more sensitive toward structural deformation and are directly helpful for determining critical damage and its location. On the other hand, acceleration data are less sensitive but more helpful for assessing global structural deformations and overall structural health assessment. The gyroscope sensor’s accuracy in predicting damage advocates its relevance but shows a complex relationship requiring deeper and more complicated analyses. Such insight can help understand the optimum number of sensing units required during the logistics and building use phases and the most suitable locations for installing sensors. However, further elaboration needs future research with this perspective in particular. Such future elaboration can also help improve the device and its performance. Moreover, the developed system is demonstrated using a wooden module for cost-effectiveness. However, further validation is needed for steel and concrete types of modules.

The developed sensing system employs state-of-the-art micro technologies, which can embed Artificial intelligence (AI) algorithms on the device. This feature allows for instantly sensing, assessing, and predicting the structural condition on the device, reducing the raw sensor data transmission and processing requirement and, hence, improving portability. The sensing system opens new research avenues for researchers by accessing detailed information on structural responses during logistic operations. It will help to understand the structural dynamics under various scenarios of module handling during logistic operations. It will help improve the structural design and the module logistics strategies to save costs and time. Also, in the future, the sensing device can be further developed to facilitate the automation of the assembly process.

## Figures and Tables

**Table 1:** Commonly used sensing technologies for structural response monitoring.

Technology	Sensors	Features
Vibration-based	Accelerometers	Global range, limited resolution, sensitive to environmental conditions and disturbances
Strain-based	Foil Strain Gauge, Piezoelectric Sensors, FBG Sensors	Local range, limited resolution, high sensitivity, sensitivity to environmental conditions, accurate damage quantification,
Guided waves	Piezoelectric Sensors	Mid-range, high sensitivity, not suitable for thick composite materials, sensitive to noise
Acoustic emission	PZT acoustic wave sensors, AE probes	Mid-range, not suitable for thick composite materials, sensitive to noise

**Table 2:** Material properties of the built module.

Materials	Elastic Modulus	Density	Poison’s Ratio
Timber Frame	14,000 MPa	750 kg/m^3^	0.18
Balsa Plywood Walls	4000 MPa	300 kg/m^3^	0.35

**Table 3:** FFT Spectrum Interquartile Range.

SU	X-Accel		Y-Accel		Z-Accel		Yaw		Roll		Pitch		Net Z-Scores
	std	3rd Q	std	3rd Q	std	3rd Q	std	3rd Q	std	3rd Q	std	3rd Q	
S1	380.89	133.82	27.80	61.47	32.30	46.68	3.22	5.54	3.25	3.23	2.06	4.60	0.83
S2	408.98	147.20	20.57	37.61	35.54	51.35	3.10	5.38	2.98	3.98	1.85	4.14	0.80
S3	383.45	123.39	17.06	39.90	35.38	87.06	3.33	5.60	3.15	4.78	1.89	3.01	0.61
S4	383.45	123.35	17.06	39.85	35.38	86.91	3.33	5.60	3.15	4.77	1.89	3.01	0.61
S5	376.62	142.55	14.02	33.62	23.22	46.66	3.30	5.44	3.25	5.05	4.76	3.30	0.97
S6	374.30	121.07	13.20	29.72	42.03	52.94	3.55	5.76	2.92	3.05	2.34	4.68	1.03
S7	381.55	120.93	15.08	32.02	22.77	53.75	3.34	5.44	2.07	5.07	1.54	3.17	0.90
S8	385.82	125.09	26.06	28.75	29.65	75.08	3.10	5.34	2.73	3.34	3.07	4.99	0.87

**Table 4:** Analyses Fusion for Locating Damages.

Category	MAW	EAW	SFH	FFT	Analyses Fusion	Impact Location			
						Front	Right	Back	Left
High	S4A_t, S6B_b, S3B_t	S4A_t, S6B_b, S3B_t	S4A_t, S3B_t	S6	S4A_t, S6B_b, S3B_t, S6	✓✓✓	✓		✓
Moderate	S7B_b, S7A_b	S7A_b, S7B_b	S6B_b, S7A_b, S7B_b	S5, S7	S7B_b, S7A_b, S5, S7	✓✓	✓	✓	✓✓
Low	S2B_t	-	S2B_t	S8	S2B_t, S8	✓		✓	✓

**Table 5:** Overall Module’s Health Impact Based on Sensor Fusion Scenarios.

Location	Right Wall	Back Wall	Left Wall	Front Wall
Strain Only Impacts				
High	9.35%	0.00%	13.55%	22.90%
Moderate	1.25%	10.89%	9.64%	0.00%
Low	1.56%	0.03%	0.00%	1.53%
Total Impact	12.15%	10.92%	23.19%	24.42%
Acceleration Only Impacts				
High	17.52%	8.91%	0.00%	8.61%
Moderate	8.65%	5.08%	0.00%	3.57%
Low	0.00%	1.81%	2.31%	0.50%
Total Impact	26.17%	15.81%	2.31%	12.67%
Gyro Only Impacts				
High	5.14%	1.81%	0.00%	3.33%
Moderate	0.00%	1.06%	2.95%	1.90%
Low	0.67%	0.00%	1.40%	2.07%
Total Impact	5.82%	2.87%	4.35%	7.30%
Equal Weighted Average Impacts				
High	7.09%	0.00%	0.94%	8.04%
Moderate	1.48%	3.85%	2.38%	0.00%
Low	0.13%	0.00%	0.62%	0.75%
Total Impact	8.70%	3.85%	3.94%	8.79%

## Data Availability

The data presented in this study are available on request from the corresponding author.

## References

[1] GovHK Modular Integrated Construction Method. https://www.info.gov.hk/gia/general/202304/26/P2023042600411.htm.

[2] Arshad H., Zayed T. (2022). Critical influencing factors of supply chain management for modular integrated construction. Automat. Constr..

[3] Zhai Y., Chen K., Zhou J.X., Cao J., Lyu Z., Jin X., Shen G.Q.P., Lu W., Huang G.Q. (2019). An Internet of Things-enabled BIM platform for modular integrated construction: A case study in Hong Kong. Adv. Eng. Inform..

[4] Wuni I.Y., Shen G.Q., Mahmud A.T. (2019). Critical risk factors in the application of modular integrated construction: A systematic review. Int. J. Constr. Manag..

[5] Luo L., Qiping Shen G., Xu G., Liu Y., Wang Y. (2019). Stakeholder-associated supply chain risks and their interactions in a prefabricated building project in Hong Kong. J. Manag. Eng..

[6] Darko A., Chan A.P., Yang Y., Tetteh M.O. (2020). Building information modeling (BIM)-based modular integrated construction risk management–Critical survey and future needs. Comput. Ind..

[7] Hsu P.-Y., Angeloudis P., Aurisicchio M. (2018). Optimal logistics planning for modular construction using two-stage stochastic programming. Automat. Constr..

[8] Luo L., Jin X., Shen G.Q., Wang Y., Liang X., Li X., Li C.Z. (2020). Supply chain management for prefabricated building projects in Hong Kong. J. Manag. Eng..

[9] Abdelmageed S., Abdelkhalek S., Hussien M., Zayed T. (2023). A hybrid simulation model for modules installation in modular integrated construction projects. Int. J. Constr. Manag..

[10] Smith I., Asiz A., Gupta G. (2007). High Performance Modular Wood Construction Systems.

[11] Godbole S., Lam N., Mafas M., Fernando S., Gad E., Hashemi J. (2018). Dynamic loading on a prefabricated modular unit of a building during road transportation. J. Build. Eng..

[12] Ditommaso R., Mucciarelli M., Ponzo F.C. (2012). Analysis of non-stationary structural systems by using a band-variable filter. Bull. Earthq. Eng..

[13] Worden K., Baldacchino T., Rowson J., Cross E.J. (2016). Some Recent Developments in SHM Based on Nonstationary Time Series Analysis. Proc. IEEE.

[14] Güemes A., Fernandez-Lopez A., Pozo A.R., Sierra-Pérez J. (2020). Structural health monitoring for advanced composite structures: A review. J. Compos. Sci..

[15] Cawley P. (2018). Structural health monitoring: Closing the gap between research and industrial deployment. Struct. Health Monit..

[16] Stratford T.J., Burgoyne C.J. (2000). The toppling of hanging beams. IJSS.

[17] Spencer B.F., Park J.W., Mechitov K.A., Jo H., Agha G. (2017). Next Generation Wireless Smart Sensors Toward Sustainable Civil Infrastructure. Procedia Eng..

[18] Fu Y., Mechitov K., Hoang T., Kim J.R., Lee D.H., Spencer B.F. (2019). Development and full-scale validation of high-fidelity data acquisition on a next-generation wireless smart sensor platform. Adv. Struct. Eng..

[19] Won J., Park J.-W., Park J., Shin J., Park M. (2021). Development of a Reference-Free Indirect Bridge Displacement Sensing System. 2021, 21, 5647. Sensors.

[20] Sarwar M.Z., Saleem M.R., Park J.W., Moon D.S., Kim D.J. (2020). Multi-metric Event-Driven System for Long-Term Wireless Sensor Operation for SHM Applications. IEEE Sens. J..

[21] Khayam S.U., Won J., Shin J., Park J., Park J.-W. (2023). Monitoring Precast Structures During Transportation Using A Portable Sensing System. Automat. Constr..

[22] Lv W., Meng F., Zhang C., Lv Y., Cao N., Jiang J. A General Architecture of IoT System. Proceedings of the 2017 IEEE International Conference on Computational Science and Engineering (CSE) and IEEE International Conference on Embedded and Ubiquitous Computing (EUC).

[23] Arduino iNEMO Inertial Module. https://content.arduino.cc/assets/st_imu_lsm6ds3_datasheet.pdf.

[24] SparkFun 24-Bit Analog-to-Digital Converter (ADC). https://cdn.sparkfun.com/datasheets/Sensors/ForceFlex/hx711_english.pdf.

[25] ESPRESSIF ESP-NOW: ESP-IDF Programming Guide. https://docs.espressif.com/projects/esp-idf/en/stable/esp32/api-reference/network/esp_now.html.

[26] Yassein M.B., Shatnawi M.Q., Aljwarneh S., Al-Hatmi R. Internet of Things: Survey and open issues of MQTT protocol. Proceedings of the 2017 International Conference on Engineering & MIS (ICEMIS).

[27] Cui J., Zhao Q., Yan G. (2019). Effective bias warm-up time reduction for MEMS gyroscopes based on active suppression of the coupling stiffness. Microsyst. Nanoeng..

[28] Bhatt D., Aggarwal P., Bhattacharya P., Devabhaktuni V. (2012). An Enhanced MEMS Error Modeling Approach Based on Nu-Support Vector Regression. Sensors.

[29] Khan A., Hammerla N., Mellor S., Plötz T. (2016). Optimising sampling rates for accelerometer-based human activity recognition. Pattern Recog. Lett..

[30] Alves V., Cury A., Cremona C. (2015). On the use of symbolic vibration data for robust structural health monitoring. Proc. Inst. Civ. Eng. Struct. Build..

[31] Kaya Y., Safak E. (2015). Real-time analysis and interpretation of continuous data from structural health monitoring (SHM) systems. Bull. Earthq. Eng..

[32] de Almeida Cardoso R., Cury A., Barbosa F. (2019). Automated real-time damage detection strategy using raw dynamic measurements. Eng. Struct..

[33] Haslbeck M., Böttcher J., Braml T. (2023). An Uncertainty Model for Strain Gages Using Monte Carlo Methodology. Sensors.

[34] Alves V., Cury A., Roitman N., Magluta C., Cremona C. (2015). Structural modification assessment using supervised learning methods applied to vibration data. Eng. Struct..

[35] Tochaei E.N., Fang Z., Taylor T., Babanajad S., Ansari F. (2021). Structural monitoring and remaining fatigue life estimation of typical welded crack details in the Manhattan Bridge. Eng. Struct..

[36] Cerna M., Harvey A.F. The Fundamentals of FFT-Based Signal Analysis and Measurement. https://www.sjsu.edu/people/burford.furman/docs/me120/FFT_tutorial_NI.pdf.

[37] Singh B.S.B., Rai S. An ML-based ERA Algorithm for Estimation of Modes Utilizing PMU Measurements. Proceedings of the 2022 3rd International Conference for Emerging Technology (INCET).

[38] Annabi-Elkadri N. (2013). Automatic Detection of Transition Zones in Tunisian Dialect. Int. J. Adv. Sci. Technol..

[39] Coverley P.T., Staszewski W.J. (2003). Impact damage location in composite structures using optimized sensor triangulation procedure. Smart Mater. Struct..

[40] Wang S., Zhang Z., Wang P., Tian Y. (2021). Failure warning of gearbox for wind turbine based on 3σ-median criterion and NSET. Energy Rep..

